# Wheat seed embryo excision enables the creation of axenic seedlings and Koch’s postulates testing of putative bacterial endophytes

**DOI:** 10.1038/srep25581

**Published:** 2016-05-06

**Authors:** Rebekah J. Robinson, Bart A. Fraaije, Ian M. Clark, Robert W. Jackson, Penny R. Hirsch, Tim H. Mauchline

**Affiliations:** 1 Rothamsted Research, West Common, Harpenden, AL5 2JQ, UK; 2 School of Biological Sciences, University of Reading, Whiteknights, RG6 6AH, UK

## Abstract

Early establishment of endophytes can play a role in pathogen suppression and improve seedling development. One route for establishment of endophytes in seedlings is transmission of bacteria from the parent plant to the seedling via the seed. In wheat seeds, it is not clear whether this transmission route exists, and the identities and location of bacteria within wheat seeds are unknown. We identified bacteria in the wheat (*Triticum aestivum*) cv. Hereward seed environment using embryo excision to determine the location of the bacterial load. Axenic wheat seedlings obtained with this method were subsequently used to screen a putative endophyte bacterial isolate library for endophytic competency. This absence of bacteria recovered from seeds indicated low bacterial abundance and/or the presence of inhibitors. Diversity of readily culturable bacteria in seeds was low with 8 genera identified, dominated by *Erwinia* and *Paenibacillus*. We propose that anatomical restrictions in wheat limit embryo associated vertical transmission, and that bacterial load is carried in the seed coat, crease tissue and endosperm. This finding facilitates the creation of axenic wheat plants to test competency of putative endophytes and also provides a platform for endophyte competition, plant growth, and gene expression studies without an indigenous bacterial background.

Endophytic bacteria reside in the internal tissue of living plants without causing symptoms of disease^1^,^2^. They are thought to be ubiquitous in all higher plants^3^ and often provide benefits for their host, including: phytostimulation^4^,^5^,^6^; biofertilisation^7^,^8^,^9^; and pathogen control^10^,^11^,^12^,^13^,^14^. These beneficial traits or products may provide novel solutions to sustainable disease control leading to quantitative and qualitative enhancement of yield^15^,^16^,^17^.

Wheat, together with rice and maize form the top three cereals grown in the world both now and for the predicted future^18^. Endophytes have been shown to provide beneficial traits in a number of other species and if these beneficial traits are to be applied to agricultural crops we need to improve our understanding of endophyte communities, colonisation routes and host interactions. The transmission of endophytes in seeds is an area of research which is only recently receiving full recognition for the role it plays in shaping microbial communities^19^. Here we investigate wheat seeds as a possible transmission route of endophytes into wheat seedlings and use our findings to develop an axenic system to study the effects of endophyte colonisation on wheat seedlings.

For an endophyte to successfully colonise the host plant, it may be beneficial to establish at an early stage when competition is at a minimum^20^. Endophytes transmitted on or in the seed would therefore appear to have a competitive advantage over later establishing endophytes^21^. In some instances this relationship between the plant and seed endophytes has evolved to the point of obligatory commensalism^22^. The establishment of a bacterial community in the spermosphere, the area immediately adjacent to a germinating seed, is shaped by seed borne bacteria^23^ which play a role in the suppression of pathogens such as fungal wilt (*Pythium* spp.)^24^. Germinating seeds are most vulnerable to pathogen invasion between 12–24 hours after imbibition^25^ and hence the early establishment of beneficial bacteria via the seed is important.

The prevalence of endophytes in seeds varies widely and can be low or non-existent^26^,^27^. Mundt and Hinkle^26^ estimated that ‘culturable’ bacterial numbers in seeds were as low as two per gram. They also noted that phytopathogens accounted for up to one quarter of the bacterial colonies found in studies of culturable seed endophytes. In a more recent study Lundberg and colleagues^28^ were unable to isolate endophytic bacteria from germinated surface sterile *Arabidopsis thaliana* seeds^28^. In contrast, seed transmission of endophytes occurs commonly in rice and appears to be important in shaping the endophyte community in the mature plant. Cultured bacterial population densities up to 3.5 ×  10^5^ CFU g^−1^ fresh tissue were found in rice seeds and 9 genera were identified by culture dependent analysis and 17 genera through culture independent analysis^29^. Hardoim and colleagues demonstrated that the aerial tissues were rapidly colonised by seed borne endophytes and that the leaf communities closely resembled seed endophyte communities^29^. Ruiza *et al.*^30^ also found endophyte densities of up to 5 ×  10^6^ CFU g^−1^ fresh tissue in rice seeds and isolated a number of genera including *Pantoea, Curtobacterium, Paenibacillus, Rhizobium* and *Microbacterium*^30^. Similarly Kaga *et al.*^20^ concluded that the rice seed is an important source of endophytes and found a number of other genera including *Bacillus*, and *Caulobacter*^20^. Transmission of endophytes from surface sterilised seed has also been demonstrated in *Eucalyptus* seeds^31^, and *Phaseolus* bean seeds^32^.

Transmission of bacteria via seeds can be separated into two pathways. The first is deposition of bacteria onto the seed surface. This transmission may not strictly be termed ‘true vertical transmission’ since the bacteria do not originate from within the host. However, transmission on the seed surface provides a rich source of bacteria for early endophytic^33^ or pathogenic^34^ colonisation of the seedling. The wheat caryopsis has a porous seed coat and a deep crease area, both of which may provide a protected cavity for harbouring bacteria. The second path of transmission is vertical transmission from the parent plant into the filial tissue. Endophytes are able to travel from the roots through to the aerial tissues via the xylem in grape vines^35^. The xylem is therefore a proposed route for endophyte entry into the caryopsis. However, in wheat there are two barriers which may prevent this; a discontinuity of the xylem at the junction between the rachilla and the pericarp^36^,^37^, and suberised cells of the chalazal layer^38^. Rice does not have the discontinuity in the xylem which is found in wheat and the structure of the chalazal cells and the transfer cells differ^39^. Thus, it is likely that the routes available for true vertical transmission found in the rice caryopsis are not present in wheat.

In this study we assessed the presence and identified which bacteria were present on seeds of wheat cv. Hereward and could further be transmitted during plant growth. We hypothesised that vertical transmission of endophytes occurs primarily in the seed coat and is absent in wheat embryos. This was confirmed through culture and molecular methods. This finding facilitated the development of an embryo derived axenic test system to screen bacterial isolates for endophytic competency.

## Results

### Recovery of bacteria from seeds and seedlings

Serial dilutions of macerated surface sterile wheat seeds (cv. Hereward) did not yield any colonies. However, colonies were recovered from seedlings derived from surface sterilised seeds grown in axenic conditions. Total endophyte densities ranged between 8.0 ×  10^3^ and 1.6 ×  10^5^ CFU g^−1^ FW. The genera recovered from the roots of 5-week old seedlings are listed in Table 1. The total culturable bacterial diversity in the seedlings was low, with only 8 genera identified at 97% similarity (genus level). Two genera together accounted for a total of 99% of the total sequence reads from colonies. The most abundant bacterial strain recovered from wheat seeds was identified as a representative of the genus *Erwinia*. This genus accounted for 88.4% of all sequence reads and was found across all biological replicates. The second most abundant strain belonged to the genus *Paenibacillus* which accounted for 10.6% of all reads and was found in two thirds of the seedlings.

### Obtaining axenic seedlings through excision of the embryo

The embryo of the wheat seed was excised and surface sterilised to eliminate the bacterial load carried in the endosperm and seed coat. Germination success for the excised embryos (85–95%) was slightly poorer than for whole caryopses (95–100%). At three weeks post germination, growth parameters for the seedlings grown from excised embryo were significantly reduced compared to the seedlings grown from whole caryopses (Fig. S1). Whole plant fresh weight was significantly lower in the seedlings from the excised embryos, reduced to 31% of the fresh weight of the caryopsis grown seedlings. Leaf height in the embryo grown seedlings was also reduced to 62% of the height of the caryopsis grown seedlings.

Endophyte recovery demonstrated that embryo excision significantly reduced or eliminated the seed bacterial load when compared with seedlings grown from the whole caryopsis (Mann Whitney U, p <  0.01) (Fig. S2). Six of nine biological replicates were sterile with no colonies recovered, and two further biological replicates recovered only 200 CFU g^−1^ FW. In contrast, colonies were invariably recovered from seedlings grown from the whole caryopsis, with a median value of 1.3 ×  10^4^ CFU g^−1^ FW.

### Culture independent confirmation of axenic seedlings

**Figure 1:**
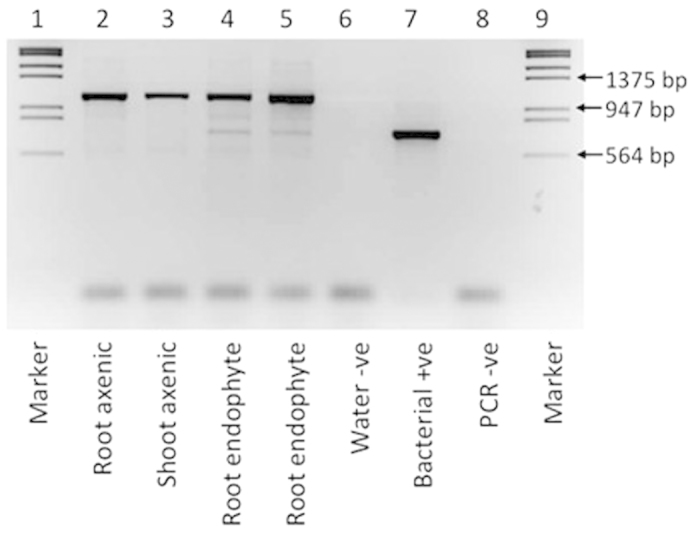
PCR amplification of bacterial 16S rRNA genes from axenic and inoculated seedlings. PCR primers 799f and 1492r were used to amplify a plant mitochondrial product (~1,100 bp) and a bacterial 16S rRNA gene product (~625 bp). Lanes 1 and 9 - molecular weight marker Lambda DNA/EcoRI + HindIII ladder; lane 2 - DNA extraction from root tissue of embryo grown seedlings; lane 3 - DNA extraction from shoot tissue of embryo grown seedlings; lanes 4, 5 - comparative seedlings with known presence of endophytic bacteria (10^6^ CFU g^−1^ FW); lane 6 - water control for the DNA extraction; lane 7 - positive bacterial control from pure culture *Variovorax paradoxus* (10^5^ CFU), lane 8 - PCR water negative control.

The presence of non-culturable endophytic bacteria in seedlings was tested using PCR primers to amplify a product from the 16S ribosomal RNA gene and a plant mitochondrial product. The DNA extraction from the whole caryopsis grown seedlings resulted in two products; a larger and more abundant product of 1,100 bp from plant mitochondrial amplification and a smaller less abundant product from bacterial 16S rRNA genes. This confirmed the presence of bacteria in the seedling as detected through plate culture. The pure culture bacterial positive control showed a strong intensity product amplified from the 16S rRNA gene at ~625 bp and both the water DNA extraction negative control and the PCR negative control showed no amplification. However, no detectable bacterial 16S rRNA gene product was amplified from the DNA extractions of culture-sterile samples of roots or shoots of embryo-grown seedlings (Fig. 1). The mitochondrial product was clearly visible but no bacterial product at ~625 bp was detected. This was in contrast to the products visible for the excised embryo seedlings inoculated with an endophytic *V. paradoxus* strain, which resulted in a visible product at ~625 bp. Extraction, PCR re-amplification and sequencing of this product confirmed its amplification from the *V. paradoxus* 16S rRNA gene, and the sequence trace was high quality indicating no detectable contamination from other bacterial strains. This indicates that bacterial endophytes are either eliminated in the embryo-derived seedlings or the bacterial load is below the PCR detection threshold.

### Confirmed re-isolation of endophytic strains from the axenic seedling system

The axenic wheat system was subsequently trialled to determine endophytic competency of a library of putative bacterial endophyte strains collected from field grown wheat in a previous study^40^ (Table 2). Neither of the negative controls, *Bacillus mycoides* nor *E. coli* K12, were isolated as endophytes from the roots or from the leaves. Both the positive controls were recovered as endophytes from the roots and the shoots. *Variovorax paradoxus* was recovered at densities greater than 1 ×  10^5^ CFU g^−1^ FW in both tissues. All putative endophytic strains tested were recovered as endophytes from the roots. Only one strain, *Cellulomonas* sp., was not recovered as an endophyte from the leaves. A number of endophytes were recovered at high densities (confluent plates, greater than 1,000 colonies) indicating colonisation of the tissue at a density greater than 1 ×  10^5^ CFU g^−1^ FW. These highly efficient colonisers include the genera *Variovorax*, *Pseudomonas*, *Stenotrophomonas*, *Flavobacterium*, *Bacillus*, and *Paenibacillus.*

## Discussion

Bacterial colonies were not recovered from surface sterilised seed macerate indicating absence or very low abundance of culturable bacteria in wheat seeds or the presence of bacterial growth inhibitors in the seed. However, germinated surface sterilised seeds grown further in sterile conditions did establish rhizosphere and endosphere bacterial populations. Seeds are very vulnerable to damage by pathogens during development, subsequent dormancy and germination and contain a number of compounds and proteins with antimicrobial activity. Antimicrobial puroindolines are found in the aleurone layer and the endosperm of wheat seeds^41^,^42^. Wheat seeds also contain high concentrations of phenolic acids^43^, which can act as inhibitors of bacterial growth. The prevalence of these antimicrobial compounds in wheat seed macerate is likely to be inhibiting recovery by culture, which should be considered when results are reported claiming sterility of seeds using culture methods.

The sequencing and identification of cultured endophyte communities recovered from the roots of wheat seedlings demonstrated a low bacterial diversity associated with the batch of wheat seeds used in this study (Table 1). Only 8 OTUs were identified at the genus level and two genera accounted for 99% of the reads, *Erwinia* (88.4%) and *Paenibacillus* (10.6%). The Gram-negative genus *Erwinia* (phylum Proteobacteria) is often associated with plant pathogens, for example in bean seeds^44^ and rice grain^45^. However, *Erwinia* has also been associated with bio-control of fungal pathogens in wheat seeds^46^,^47^. The wheat seeds used in this study had 95–100% germination success and the seedlings appeared to have healthy growth, indicating that the *Erwinia* strain isolated is not pathogenic, although further confirmation is needed. The other prevalent genus was *Paenibacillus*, from the Firmicute phylum. Many Gram-positive bacteria (including Firmicutes) are able to form endospores which are likely to contribute to the survival of the bacteria for long periods in the seed.

We hypothesised that in wheat the barriers in the xylem and the chalazal cells might limit true vertical transmission from the maternal to filial tissue. Endophytic and pathogenic bacteria are commonly associated with the seed coat and rarely associated with the embryo. In a study of *Salmonella* infections of alfalfa seed, Charkowski *et al.*^48^ concluded that wrinkled seeds were harder to sterilise than smooth seeds, suggesting that convolutions in the seed coat are a major site of colonisation^48^. Darrasse *et al.*^49^ studied *Xanthomonas* infection of bean seeds and concluded that surface transmission from floral tissue is a more permissive pathway of infection than the vascular pathway into the seed^49^. Attachment to the seed surface allows pathogens to evade the plant defence system and the seed becomes a passive carrier. In the few cases where bacteria are associated with the embryo it is linked to heavy pathogen infection and necrosis and browning of the embryo^50^,^51^. Wheat has a porous seed coat and a deep crease tissue which may act to harbour bacteria even after surface sterilisation of the whole seed.

We excised and germinated wheat embryos to test the hypothesis that few bacteria are transmitted in the wheat embryonic tissue. The seedlings from excised embryos had significantly reduced or eliminated bacterial endophytes; the majority of these plants were shown to be sterile through culture-dependent and independent methods, indicating that few bacteria are vertically transmitted. It seems probable in light of plant defence and evolutionary processes that the wheat embryo generally exists as a sterile entity. The main bacterial load carried in the seed coat or the endosperm can originate from passive access or through penetration of ovary wall and/or floral parts.

Although the PCR primers 799f and 1492r have been successfully used in culture independent endophyte enrichment studies^52^ the co-amplification of the abundant mitochondrial product limits the detection of the bacterial endophytes. We found that these primers can be used to detect the presence of an endophytic *Bacillus* sp. at an approximate density of 10^6^ CFU g^−1^ tissue (determined via culture). However, the product is very faint compared to the wheat mitochondrial product and the competitive amplification of the mitochondrial and bacterial target DNA may mean that bacterial endophytes exist below the detection limit for these primers. Further studies would be needed to determine the lowest sensitivity of the primers for detecting bacterial DNA in different plant backgrounds. Designing phyla specific primers to amplify bacteria from a wheat background may improve sensitivity.

Many bacterial strains have the ability to live as an endophyte for some part of their life cycle; however, not all bacterial strains have the traits required for competent endophytic colonisation of plants^53^. We used a root drench inoculation method to test for endophyte competency. Unlike other methods such as injection of the bacterial endophyte into the stem^54^, we are attempting to exclude opportunistic endophytes which can enter the plant through damage by herbivores or by mechanical breakage. Neither of the negative controls, *E. coli* K12 and *Bacillus mycoides,* were recovered as endophytes in the roots or the leaf tissue, whereas the positive control strains *Pseudomonas fluorescens* SBW25 and *Variovorax paradoxus*, both known wheat endophytic bacteria, were recovered as endophytes from both the roots and the leaves. These results demonstrate that surface sterilisation is sufficient to remove rhizosphere colonising bacteria (*E. coli* and *Bacillus mycoides*), but is not so harsh that it penetrates the tissue and kills the internal endophytes (*Pseudomonas* SBW25 and *Variovorax paradoxus*). It will also be interesting in future studies to determine what proportion of the bacterial inoculant colonises the endosphere and how much proliferates in the rhizoplane, and more general rhizosphere.

A notable finding is that the axenic seedling system, despite being gnotobiotic without indigenous endophytes or the chemical defences found in the endosperm, does not facilitate all bacterial strains to become endophytic. Competent rhizosphere colonisers are not automatically competent endosphere colonisers demonstrating that bacterial endophytic colonisation is not a passive process. We demonstrate for the first time that an axenic seedling system can be used to distinguish competent endophytes from rhizosphere colonisers in wheat.

Nearly all the putative endophytes tested were recovered as endophytes from both the roots and the leaves. This presence of the endophyte in a different tissue (leaves vs. roots) to the initial inoculation provides strong evidence that the endophytes have colonised the internal tissue of the seedlings and that the strains are truly endophytic. Of the inoculated putative endophytes only one, *Cellulomonas* sp., was not recovered from the leaves. It is interesting that this strain was originally recovered only from the leaf tissue and was not found in the roots. It may therefore indicate that this strain is not mobile in the xylem and colonisation of the leaf tissue may have occurred after rain splash or airborne dispersal, or through wound entry. Most endophytes were recovered at 10^3^ to 10^4^ CFU g^−1^ FW. However a few returned confluent plates with greater than 1,000 colonies, equivalent to greater than 10^5^ CFU g^−1^ FW. This indicates highly efficient colonisation by these endophytes. A number of studies report a decrease in bacterial densities as the plant matures^55^,^56^. It would be interesting to see whether these highly efficient colonisers are able to maintain such high densities in the face of competition from other endophytes and as the plant matures.

In conclusion, this study demonstrates that wheat seeds carry a bacterial load. However, this load is not detectible by culture of seed macerate due to low abundance and/or bacterial growth inhibitors. Further growth of surface sterile seed in sterile conditions revealed that the diversity of bacteria carried in the seed is relatively low, but these bacteria are able to rapidly establish endophytic populations in the developing seedling. We found endophyte densities could reach 1.6 ×  10^5^ CFU g^−1^ FW in the roots after 5 weeks post imbibition. This provides support for the use of microbial seed coating to promote the establishment of beneficial organisms in seedlings. The bacteria do not have to exist as endophytes within the seed in order to rapidly colonise as endophytes in the seedling. Dissection of the wheat seed to remove the seed coat and endosperm and then subsequent surface sterilisation and germination of the embryo demonstrated that the embryo generally exists as a sterile entity, and that true vertical transmission into the embryonic tissue is not common in wheat. It is likely that the seed coat, the crease tissue and the endosperm carry the majority of the bacterial load on wheat seeds. Using the axenic wheat system we were able to distinguish endophytic and non-endophytic bacterial strains, and to confirm that 24 putative endophytic strains were competent endophytes colonising the roots and 23 of these strains were also able to colonise the shoot tissue. This novel axenic seedling system in wheat has potential for a variety of studies to investigate endophyte competition, measurements of plant growth parameters with endophyte inoculations, and to study host responses (plant gene expression), without the background of indigenous endophytes in wheat. It is likely that that this system can be extended to other crop species.

## Methods

### Seed material

Wheat seeds (cv. Hereward) were harvested from field grown plants at Rothamsted Research in 2011. Seeds were stored for two years in a controlled environment at 15 °C. Before imbibition whole seeds were surface sterilised with 70% ethanol for 10 minutes, 1.5% active chlorine for 1 hour, and rinsed five times in sterile distilled water (SDW). Seeds were imbibed overnight in SDW at 4 °C before maceration or germination.

### Bacterial endophyte isolation from wheat seeds and seedlings

In order to determine if culturable bacteria were present in wheat seeds, surface sterilised wheat seeds were macerated and plated onto bacterial growth medium. Six replicates of three wheat seeds were surface sterilised and macerated with a pestle and mortar in 1 ml SDW per 0.1 g plant material. Serial dilutions of the macerate (0.01 g tissue and 0.001 g tissue) were plated onto 1/10^th^ dilution tryptic soy broth (TSB; Oxoid) agar in triplicate and incubated at 28 °C for 6 days.

Additionally, surface sterilised wheat seeds were germinated and grown in aseptic conditions to determine whether endophytic bacteria were subsequently detected in the seedlings. The surface sterilised seeds were germinated on 4.4 g l^−1^ Murashige and Skoog plant growth medium (Sigma MS basal salt mixture) supplemented with sucrose (3% w/v) and agar (0.8% w/v). Plants were grown in a growth chamber (Sanyo MLR 350) with a 20 °C 16 h photoperiod under fluorescent light with photon flux of 150 μ mol m^−2^ s^−1^.The seedlings were grown for 5 days until approximately 1 cm shoot and 1.5 cm roots had developed. Two Magenta® GA-7 Plant culture boxes (Magenta, Chicago, USA) were joined with a connecting unit, with the base box containing 175 g of 50:50 gravel: vermiculite (v/v) mix. The Magenta box unit with growth medium was twice autoclaved (123 °C for 15 min) with a period of 48 h between autoclaving. Three seedlings were transplanted under sterile conditions into each Magenta box unit (one biological replicate), watered with 50 ml SDW and placed into the plant growth chamber until 5 weeks post imbibition at which point 3 to 5 leaves had unfolded. Fresh weight (FW) and primary leaf height measurements were taken for seedlings.

The roots and leaves from each biological replicate were processed independently to isolate the endosphere. Surface sterilisation was performed with agitation in 2% active chlorine for 1 minute and three rinses in SDW. For a stringent surface sterility control, the total final rinse (from 0.15 g FW tissue) was centrifuged at 3,000 ×  g for 10 minutes, the pellet suspended in 100 μ l SDW, and cultured as for bacterial recovery. If fewer than 10 colonies were recovered from the total final rinse then surface contamination was considered minimal and the sample endophyte enriched. The plant sections were then macerated and aliquots plated as for the recovery of bacteria from the seeds. Colony counts were taken from all plates and total bacterial cultures from each plate were collected by suspending all colonies in 2 ml SDW and freezing at − 20 °C until further use.

### Bacterial identification using 16S rRNA gene sequencing

For identification of all cultured bacterial isolates a DNA extraction of bacterial suspensions was performed using the Sigma Aldrich GenElute bacterial DNA extraction kit. All procedures followed the manufacturer’s instructions and reactions were carried out in 2 ml micro-centrifuge tubes at 16,000 rpm. DNA was eluted in 50 μ l SDW, quantified using a Nanodrop (ND-1000 Spectrophotometer, Labtech) and stored at − 20 °C. Extracted DNA was subjected to high throughput amplicon sequencing of a fragment of the 16S rRNA gene. Paired-end sequencing was performed on an Illumina MiSeq platform using the bacterial/archaeal forward primer 515F (GTGCCAGCMGCCGCGGTAA) and reverse primer 806R (GGACTACHVGGGTWTCTAAT). After removal of primer sequences 252 bp fragments were analysed using a 16S rRNA gene profiling analysis pipeline developed in Qiime^57^. The amplicons were filtered for quality, singletons were removed, chimeras were screened and removed and taxonomy was assigned at the species and genus level.

### Embryo excision

The wheat seed is formed of multiple tissue types: primarily consisting of the seed coat, the endosperm and an embryo. Excision of the embryo from the endosperm and the seed coat eliminates any bacteria adhering to the seed coat or present in the crease tissue of the wheat seed. All work took place in a laminar flow hood under sterile conditions. Surface sterilised imbibed wheat seeds were rinsed in fresh SDW and the embryo was carefully excised from each seed by cutting through the scutellum, the connective tissue between the endosperm and the embryo. The tissue around the embryo was then further trimmed close to the embryo to minimise any traces of seed coat. The excised embryo was placed in 95% ethanol for 30 seconds then thoroughly rinsed in SDW. The excised embryos were grown as for the whole seeds and seedlings were processed as described above for recovery of culturable bacterial endophytes.

### Culture independent analysis of embryo grown seedlings

Root and shoot tissue macerate from surface-sterile embryo derived 5 week old seedlings were also processed for culture independent analysis of endophytic bacteria. DNA from 0.1 g FW of tissue was extracted using the QIAGEN DNeasy plant DNA extraction kit. The root tissue of three embryo derived seedlings inoculated with an endophytic *Variovorax paradoxus* were included as a positive control for DNA extraction. The abundance of the *V. paradoxus* in the seedling root tissue was estimated to be approximately 10^6^ CFU g^−1^ plant tissue based on dilution plating onto 1/10^th^ TSB agar. Root tissue from 5 week old seed-grown seedlings were also extracted. A water extraction was performed as a control for the sterility of the DNA extraction kit. A further positive control of approximately 10^5^ cells of pure culture *V. paradoxus* was included for DNA extraction. Extraction was carried out as per the manufacturer’s instructions with a final elution of DNA in 50 μ l.

The PCR primers 799f and 1492r, which had been designed to amplify bacterial DNA while excluding amplification of plant chloroplast DNA, were chosen to amplify a product from the 16S ribosomal RNA gene of any bacteria in the seedlings^58^. PCR amplification was carried out in 25 μ l reaction volumes containing the following reagents: 1 ×  Bioline PCR buffer, 2 mM MgCl_2_, 1 μ M each primer, 200 μ M each deoxynucleoside triphosphate, 1.25 U Bioline *Taq* polymerase, and 1 μ l of DNA template. The PCR protocol for amplification of the products was as follows: initial denaturation 95 °C for 3 min, then 30 cycles of 94 °C for 30 sec, 53 °C for 1 min, 72 °C for 1 min and a final extension at 72 °C for 7 min. PCR products were run on 1.5% agarose gels in 1 ×  TBE at 5 V/cm for 1.5 hours to separate the product. The gel was viewed and photographed under UV light. Amplicons from the control sample with *Variovorax paradoxus* were purified and sequenced with the forward and reverse PCR primers (MWG Eurofins) to confirm amplicon identity.

### Testing a form of Koch’s postulates for putative wheat endophytes using the axenic seedling system

Results indicated that surface sterilisation of embryos excised from wheat seeds results in axenic wheat seedlings. This created the opportunity to use the axenic seedling system as a screening method to determine the endophytic capability of bacterial isolates. Axenic seedlings were obtained from excised embryos as described above. Prior to transfer of germinated seedlings into the Magenta box units the growth medium was inoculated with a suspension of fresh bacterial culture. Bacterial culture was obtained as follows: 48 hours growth in 1/10^th^ tryptic soy broth at 28 °C, pelleted by centrifugation at 3,000 ×  g for 10 minutes, rinsed and re-suspended in 50 ml sterile water to an OD_600_ of 0.2 and a cell density of approximately 5 ×  10^9^ CFU ml^−1^. *Bacillus mycoides* (isolated from soil) and *E. coli* K12 were inoculated as non-endophytic controls. *Pseudomonas fluorescens* SBW25 and *Variovorax paradoxus* were inoculated as endophytic positive controls. A selection of putative endophytic strains previously isolated from wheat plants grown at the Rothamsted Farm, 2011 were also tested for their endophytic colonisation ability^40^. Bacteria were identified through amplification and sequencing of the 16S rRNA gene using the bacterial primers MF (5′ -CCTACGGGAGGCAGCAG)^59^ and 1389r (5′ -ACGGGCGGTGTGTACAA)^60^,^61^. Three axenic seedlings were placed onto the surface of the inoculated medium and grown as described for a further three weeks before harvesting for re-isolation of endophytes. The inoculated strain was considered endophytic if more than 10 colonies were recovered from the surface-sterilized roots; a detection threshold equivalent to 1.0 ×  10^3^ CFU g^−1^ FW tissue. If fewer than 10 colonies were recovered, the re-inoculation and re-isolation was repeated to a total of three replicates (each replicate used 3 seedlings in an experimental unit). If each of three replicates recovered less than 10 colonies, the inoculant was considered to be non-endophytic. Identities of recovered bacteria were assigned through partial sequencing of the 16S rRNA gene as described above to confirm whether they were identical to the inoculated strain.

## Additional Information

**How to cite this article**: Robinson, R. J. *et al.* Wheat seed embryo excision enables the creation of axenic seedlings and Koch's postulates testing of putative bacterial endophytes. *Sci. Rep.* **6**, 25581; doi: 10.1038/srep25581 (2016).

## Supplementary Material

Supplementary Information

## Figures and Tables

**Table 1:** Identity and relative abundance of cultured endophytes recovered from roots of wheat seedlings from surface sterilised seeds grown in sterile conditions.

Identification to genus level	Phylum or group	Relative abundance (% of reads)^1^	Colonisation incidence (%)^2^
*Erwinia* sp.	γ -proteobacteria	88.4	100.0
*Paenibacilus* sp.	Firmicutes	10.6	66.7
*Bacillus* sp.	Firmicutes	0.7	83.3
*Pedobacter* sp.	Bacteroidetes	0.3	50.0
*Arthrobacter* sp.	Actinobacteria	< 0.1	100.0
*Pseudomonas* sp.	γ -proteobacteria	< 0.1	83.3
*Sinorhizobium* sp.	α -proteobacteria	< 0.1	83.3
*Flavobacterium* sp.	Bacteroidetes	< 0.1	50.0

Data obtained from 18 seeds (6 biological replicates of 3 seeds).

^1^Of a total 142,252 16S rRNA gene sequence reads.

^2^% of samples within which the endophyte was recovered.

**Table 2:** Confirmed re-isolation of endophytic strains from 4-week old wheat seedlings.

Inoculated strain ref.	Accession number	OTU identity (to genus or species level)	Recovered as an endophyte in tissue^1^	
			Roots	Shoots
− ve Control^2^		*Bacillus mycoides*	No	No
− ve Control^2^		*E. coli* K12 JM109	No	No
+ ve Control^3^		*Pseudomonas fluorescens* SBW25	Yes	Yes
+ ve Control^3^		*Variovorax paradoxus*	Yes ★	Yes ★
M275	KJ649709	*Brevundimonas* sp.	Yes	Yes
J265	KJ649711	*Devosia* sp.	Yes	Yes
J334	KJ649712	*Rhizobium* sp.	Yes	Yes
J195	KJ649714	*Duganella* sp.	Yes	Yes
M416	KJ649716	*Variovorax boronicumulans*	Yes ★	Yes
J220	KJ649718	*Pseudomonas* sp.	Yes ★	Yes ★
J231	KJ649721	*Pseudomonas* sp.	Yes	Yes
J278	KJ649722	*Serratia* sp.	Yes	Yes
M9	KJ649723	*Stenotrophomonas* sp.	Yes ★	Yes
M126	KJ649724	*Aeromicrobium* sp.	Yes	Yes
M123	KJ649725	*Agreia* sp.	Yes	Yes
J289	KJ649726	*Agromyces* sp.	Yes	Yes
M386	KJ649727	*Arthrobacter* sp.	Yes	Yes
M75	KJ649728	*Cellulomonas* sp.	Yes	No
J370	KJ649729	*Curtobacterium* sp.	Yes	Yes
J263	KJ649732	*Microbacterium* sp.	Yes	Yes
J428	KJ649731	*Microbacterium* sp.	Yes ★	Yes
M49	KJ649733	*Microbacterium* sp.	Yes	Yes
M322	KJ649734	*Plantibacter* sp.	Yes	Yes
M382	KJ649735	*Rhodococcus* sp.	Yes	Yes
M21	KJ649737	*Flavobacterium* sp.	Yes ★	Yes
J257	KJ649738	*Pedobacter* sp.	Yes ★	Yes
J360	KJ649739	*Bacillus simplex*	Yes ★	Yes
J367	KJ649740	*Paenibacillus* sp.	Yes ★	Yes ★

Each replicate sample comprised of 3 seedlings grown in a Magenta pot unit. If the strain was not recovered from any of three replicates, the strain was considered non-endophytic.

^★^Strains recovered at a density greater than 1 ×  10^5^ CFU g^−1^.

^1^Putative endophytic strains were isolated from wheat plants at Rothamsted Research 2011.

^2^*Bacillus mycoides* and *E. coli* K12 were inoculated as negative controls (non-endophytic strains).

^3^*Pseudomonas* SBW25 and *Variovorax paradoxus* were included as positive controls (known endophytic strains).
