# Supplementary material for: Wheat seed embryo excision enables the creation of axenic seedlings and Koch’s postulates testing of putative bacterial endophytes
Source: Sci Rep. 2016 May 6;6:25581. doi: 10.1038/srep25581 (PMC4858700; doi:10.1038/srep25581)
Supplement: Supplementary Information [file srep25581-s1.pdf]

Wheat seed embryo excision enables the creation of axenic seedlings and Koch's postulates testing of putative bacterial endophytes

Rebekah J Robinson, Bart A Fraaije, Ian M Clark, Robert W Jackson, Penny R Hirsch & Tim H Mauchline

Supplementary Figure 1A and 1B

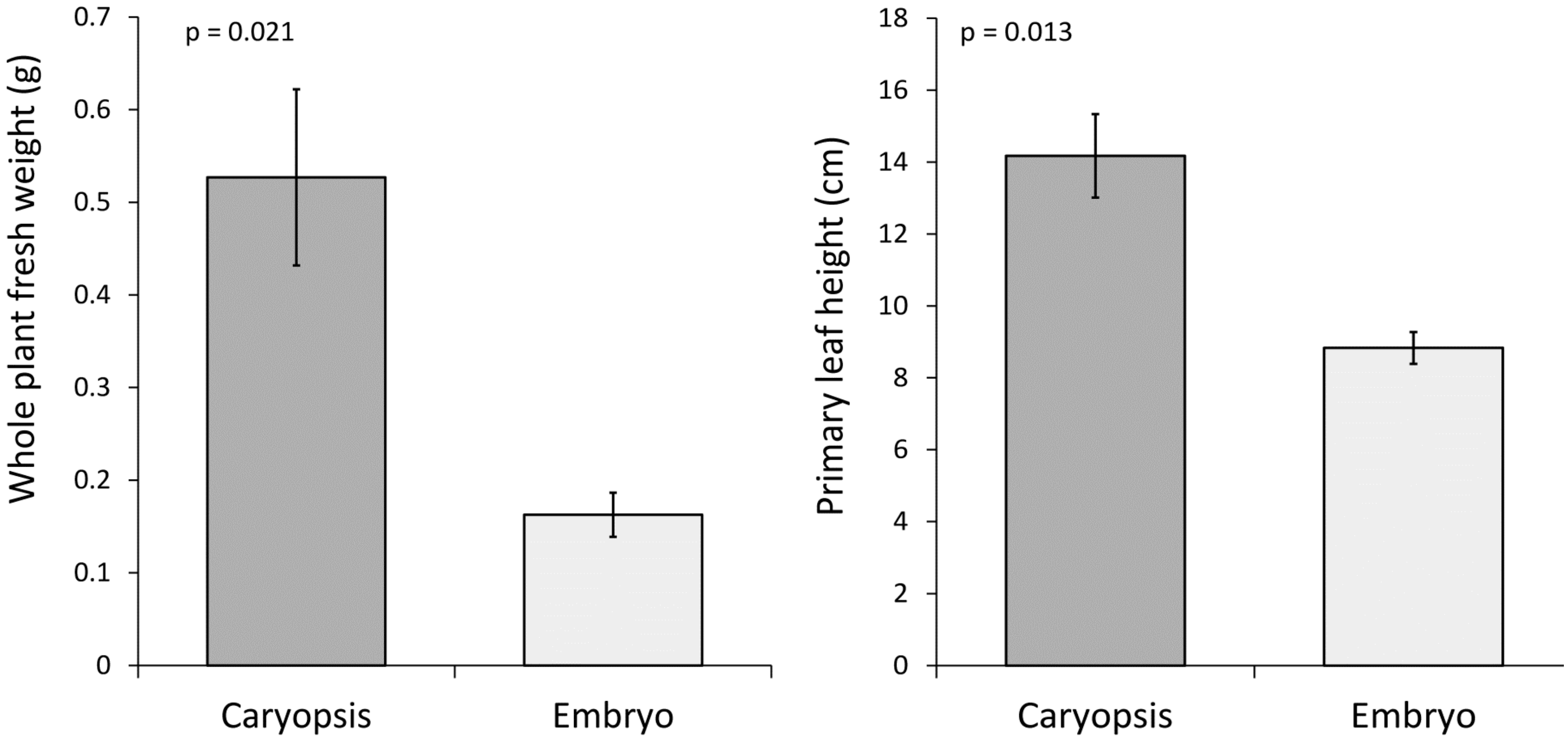

Supplementary Figure 1 Comparative growth of wheat seedlings grown from whole caryopses and excised embryos. A. Mean whole plant fresh weight. B. Mean primary leaf height. Mean values are calculated from 3 biological replicates (9 seedlings) at 3 weeks post imbibition. Significant differences are indicated with star ($p < 0.05$, two sample t-test). C. Growth at 3 weeks post imbibition i) Seed grown seedlings ii) Embryo grown seedlings

Wheat seed embryo excision enables the creation of axenic seedlings and Koch's postulates testing of putative bacterial endophytes

Rebekah J Robinson, Bart A Fraaije, Ian M Clark, Robert W Jackson, Penny R Hirsch & Tim H Mauchline

Supplementary Figure 2

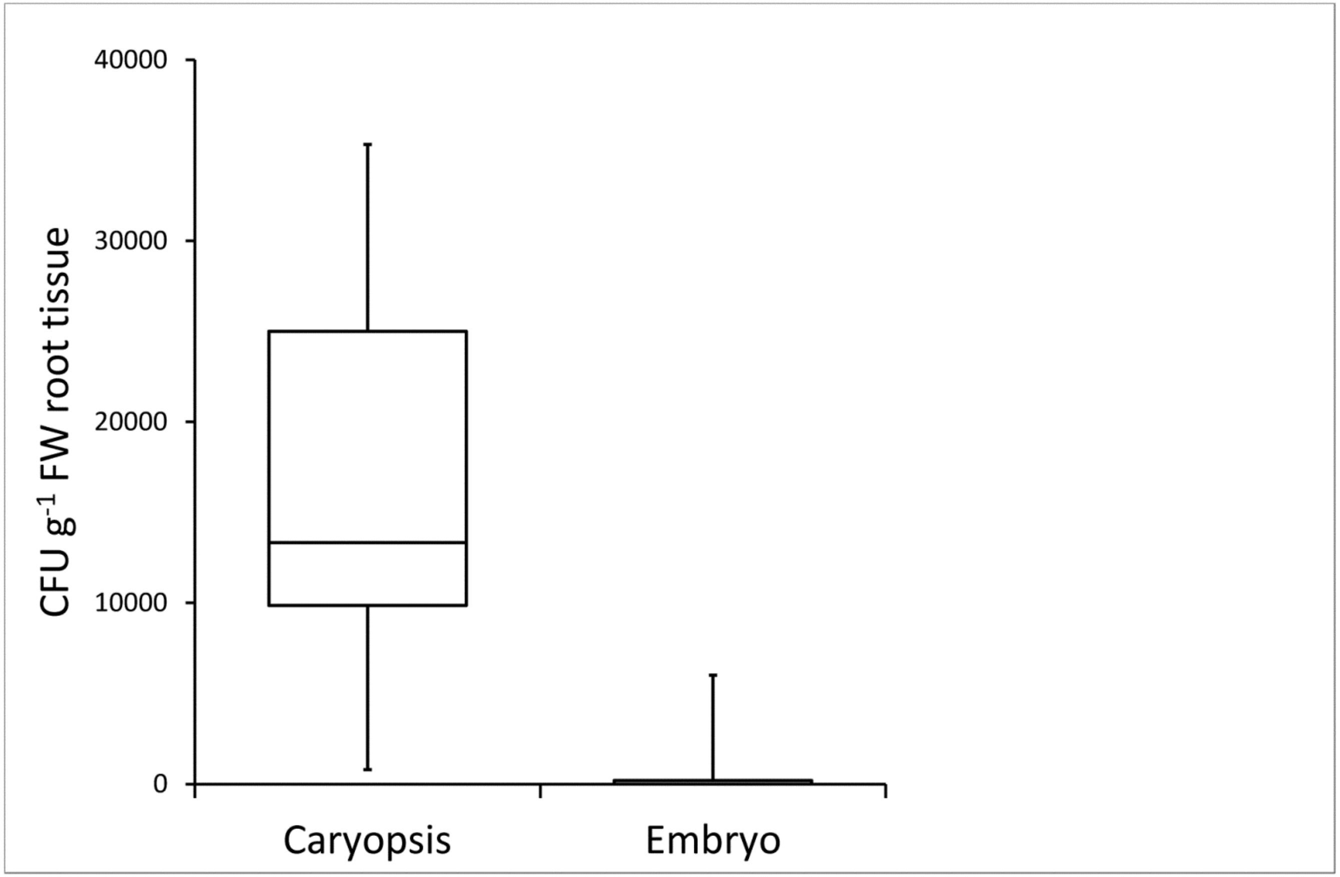

Supplementary Figure 2 Abundance of endophytes recovered from surface sterile root tissue of wheat seedlings grown from whole caryopses and excised embryos. Endophyte abundance measured in 9 biological replicates (3 seedlings in each biological unit). Box limits indicate Q1, median and Q3 values; whiskers indicate maximum and minimum values. Mann Whitey U, $p < 0.01$.
